# Intra-patient comparison of parietal pleural biopsies by rigid forceps, flexible forceps and cryoprobe obtained during medical thoracoscopy: a prospective series of 80 cases with pleural effusion

**DOI:** 10.1186/s12890-016-0258-5

**Published:** 2016-07-07

**Authors:** H. Wurps, N. Schönfeld, T. T. Bauer, M. Bock, C. Duve, R. Sauer, T. Mairinger, S. Griff

**Affiliations:** Department of Respiratory Medicine, Lungenklinik Heckeshorn, HELIOS Klinikum Emil von Behring, Berlin, Germany; Department of Pneumology and Institute of Pathology, HELIOS Klinikum Emil von Behring, Berlin, Germany

**Keywords:** Medical thoracoscopy, Cryobiopsy, Parietal pleura, Rigid forceps biopsy, Flexible forceps biopsy

## Abstract

**Background:**

There is only few data available on the use of cryotechnique during medical thoracoscopy.

**Methods:**

Medical thoracoscopy was performed in consecutive patients with pleural effusion. Prospectively, biopsies were taken by rigid forceps, flexible forceps and cryoprobe. Specimen size, depth and diagnostic yield were compared.

**Results:**

80 Patients were included. 408 biopsies were taken (205 rigid biopsies, 104 flexible biopsies, 99 cryobiopsies). Mean surface area of rigid biopsies was 22.6 ± 20.4 mm^2^ (flexible biopsies: 7.1 ± 9.3 mm^2^, cryobiopsies: 14.4 ± 12.8 mm^2^). Rigid biopsies were significantly larger than cryobiopsies (*p* < 0.001) and flexible biopsies (*p* < 0.001), crybiopsies were significantly larger than flexible biopsies (*p* < 0.01). A deep biopsy containing fatty tissue was harvested in 63 % of rigid biopsies (cryobiopsy: 49.5 % flexible biopsy: 39.5 %). In 79/80 cases (98.7 % 95 % CI cannot be calculated) a diagnosis was obtained by rigid biopsy (cryobiopsy: 73/80 cases (91.3 % 95 % CI 86.0 – 96.5 %), flexible biopsy: 74/80 cases (92.5 % 95 % CI 88.6 – 97.4 %)). Diagnostic yield achieved with cryobiopsies was inferior to the yield of rigid biopsies (Difference: 12.7 %), but non-inferior to flexible biopsies (Difference: 6.5 %).

**Conclusion:**

Cryobiopsies in medical thoracoscopy are safe with high diagnostic yield, non-inferior to flexible biopsies with increased tissue quantity and quality. Cryotechnique can develop an important role in medical thoracoscopy in the near future when rigid thoracoscopy is not available.

## Background

Medical thoracoscopy in rigid and in semi-rigid technique is an efficient and safe procedure in patients with exudative pleural effusion of unknown origin. Biopsy specimen taken during semi-rigid-thoracoscopy are smaller than biopsies taken by rigid forceps but the diagnostic accuracy is said to be similar [1–11]. An advantage of semi-rigid thoracoscopy is flexibility of the endoscope; yet the flexible forceps is smaller and less stable compared to the rigid forceps. Therefore, it is still under debate whether flexible pleural forceps biopsies have the same diagnostic potential as biopsies harvested with rigid forceps.

Cryotechnique was introduced as early as 1968, at first for the therapeutic management of airway diseases [12]. Since then and especially in the last ten years the use of cryotechnique has been established as a routine procedure in bronchoscopy for diagnostic and interventional therapeutic use [13–20]. No increase of complications has been described [21–24]. Furthermore, in diagnostic series it could be demonstrated that central and pulmonary tissue samples were larger and better preserved compared to forceps biopsies [25, 26].

The use of cryotechnique in thoracoscopy has been initially described in 1989 [27], and an analgetic advantage was noted 30 years later [28]. Recently, an article describing the feasibility of cryotechnique in medical, semi-rigid thoracoscopy in fifteen patients with exsudative pleural effusion was published [29]. In this series, cryotechnique was shown to be efficient and safe without major complications.

The aim of this prospective study was to compare thoracoscopically obtained parietal pleural biopsies by rigid forceps and flexible forceps to cryobiopsies and to document the non-inferiority of cryotechnique in this procedure.

## Methods

### Patients

This prospective study was performed at a tertiary respiratory care center (Lungenklinik Heckeshorn) between 2012 and 2014. All consecutive patients with exsudative pleural effusion of unknown etiology with indication for medical thoracoscopy were eligible for this study. Prior to the thoracoscopy and study enrollment, all patients were informed and written consent was obtained. The study was approved by the medical ethics committee of the Charité – Universitätsmedizin Berlin (Ethikkommission, Ethikausschuss 4 am Campus Charité – Mitte).

### Thoracoscopy and forceps techniques

Medical thoracoscopy with rigid single-port-of-entry technique was performed in the endoscopy suite under local anesthesia and sedation as described elsewhere [30]. All procedures were performed using a rigid thoracoscope (11 mm, Storz, Tuttlingen, Germany). To define the point of entry into the pleural cavity, an ultrasound was carried out followed by the introduction of a pneumothorax under fluoroscopic guidance. Under direct vision with the thoracoscope, all pleural fluid was removed and the pleural cavity was inspected. Afterwards, specimen were taken with rigid forceps (3 mm, Storz, Tuttlingen, Germany), flexible forceps (2.8 mm, Boston Scientific Radial Jaw, Natick, MA, USA) and cryoprobe (2.4 mm, Erbokryo CA, Erbe, Tübingen, Germany) in random order. Cryotechnique takes advantage of the Joule-Thompson-Effect (rapid gas release with high flow induces low temperature up to -77° Celsius); this leads to freezing and adhesion of specimen and parietal pleura. At last, the attached tissue was extracted together with the cryoprobe. In cases of obvious malignancy a pleurodesis with talcum poudrage was performed. After the procedure, a chest tube was placed into the pleural cavity.

### Morphometrical and morphologic analysis of thoracoscopic biopsies

All biopsies were processed conventionally by serial sectioning of at least 12 Hematoxylin-eosin (HE) stained section steps as it refers to be the standard procedure analyzing pleural biopsies in histological routine as described elsewhere [25, 26]. Serial sectioning is therefore used to avoid incomplete sectioning of particles in order to provide a valid histopathological diagnosis. All biopsies were subsequently surveyed regarding size and quality. The pathologist was not blinded regarding to the technique used to obtain the biopsy.

The HE stained slides were therefore scanned by a ZEISS-MIRAX Midi Slide scanning system using the Mirax Viewer Image Software Version 1.12 (Zeiss Microimaging, Oberkochen, Germany and 3D Tech, Budapest, Hungary). Regarding biopsy size the total area was measured by interactive circling of the largest biopsy section of each serial. All areas were calculated automatically and provided in mm^2^. Biopsy quality was determined by tissue depth. A thoracoscopic biopsy specimen including fatty tissue of the thoracic wall was considered to be a deep biopsy and therefore of high quality.

### Statistical analyses

For data analyses a statistical software (Statistical Package for Social Sciences, Version 22.0; SPSS, Chicago, IL, USA) was used on a Windows XP operating system (Microsoft; Redmond, WA, USA). Results were expressed as frequencies or as mean ± SD. The multiple comparison of surface area of the three methods (rigid, flexible, and cryobiopsy) were performed by ANOVA with post-hoc Bonferroni correction.

The following method was used to weigh observed differences between diagnostic success rates to establish a histopathological diagnosis. Two-sided 95 % confidence intervals (CI) for single proportions were calculated according to a standard formula (CI = p ± Z_α/2_ × √[(p × q)/n]) for all proportions (cases diagnosed (x) over all cases (n)) when x – n ≥ 5. We used this statistical method to assume non-inferiority for the differences in success rates. Non-inferiority was assumed if the difference of the lower limit of this confidence interval of the inferior method was not larger than 10 % compared to the highest observed success rate (assumed this to be higher than 90 %).

The significance level of the analyses was set to 5 %, and exact *p* values were reported were appropriate.

## Results

Eighty patients with a mean age of 67.5 ± 13.5 years were included in the study. For each patient, three to four biopsies by rigid forceps were taken. Due to the duration of the procedure, one to two cryobiopsies were taken as well as one to two biopsies by flexible forceps. Altogether, 408 biopsies were taken and analyzed (205 biopsies by rigid forceps, 104 biopsies by flexible forceps, and 99 biopsies by cryoprobe).

**Fig. 1:**
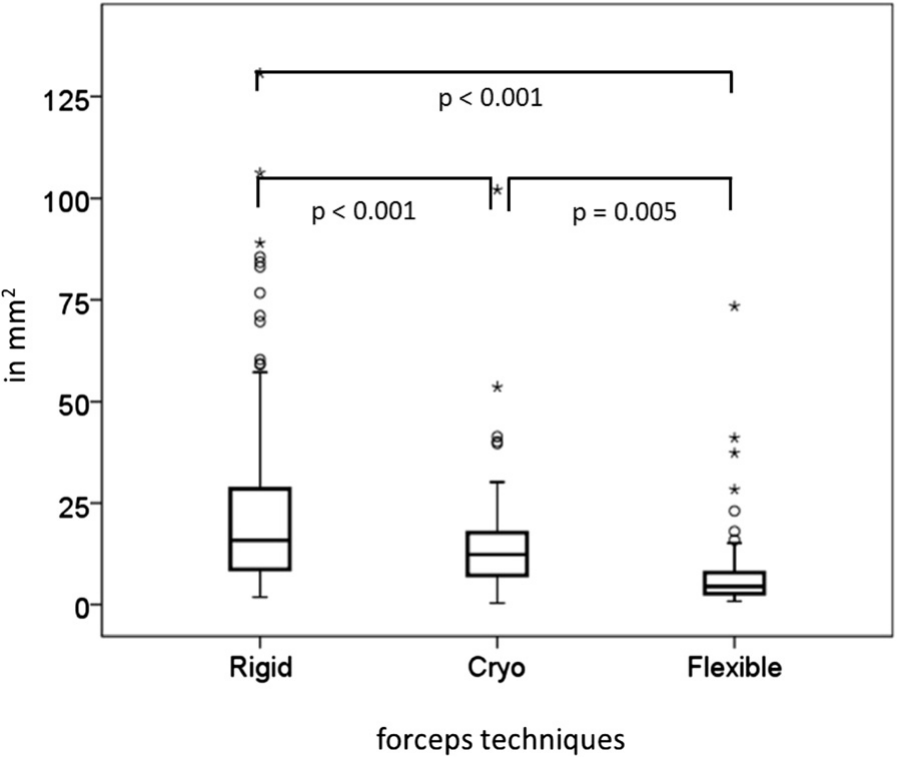
Total area of specimens taken by rigid forceps, flexible forceps and cryobiopsy (in mm^2^, p-values represent results of ANOVA with Bonferroni correction). Boxplots are shown, outliers (values that are between 1.5 and 3 times the interquartile range) are represented by circles beyond the whiskers. Extreme values (values that are more than 3 times the interquartile range) are represented by asterisk beyond the whiskers

The mean surface area of biopsies taken with the rigid forceps was 22.6 ± 20.4 mm^2^, with the flexible forceps 7.1 ± 9.3 mm^2^, and with the cryoprobe 14.4 ± 12.8 mm^2^ (Fig. 1). Rigid forceps biopsies were significantly larger than samples taken by cryoprobe (*p* < 0.001) and flexible forceps (*p* < 0.001). Biopsies by cryoprobe were significantly larger than flexible forceps biopsies, too (*p* < 0.01; one-way ANOVA, with Bonferroni correction).

A deep biopsy containing fatty tissue was obtained in 63 % of the rigid forceps biopsies, in 49.5 % of the samples harvested with the cryoprobe and in 39.5 % of the biopsies by flexible forceps (see Table 1).

**Table 1:** Comparison of forceps techniques concerning number of biopsies and inclusion of fatty tissueᅟ

	Number of biopsies	Positive for fat tissue	Negative for fatty tissue	Biopsies incl. fatty tissue (%)
Rigid forceps biopsy	205	129	76	62.9
Flexible forceps biopsy	104	41	63	39.4
Cryoprobe biopsy	99	49	50	49.5

In 66 out of all 80 cases (83 %) a deep biopsy was obtained by using the combination of all three described methods.

### Histopathologic diagnoses

In the histopathological work-up, 43/80 malignant (54 %) and 37/80 non-malignant (46 %) diagnoses were found (see Table 2).

**Table 2:** Overview of histopathological diagnosis and the number of cases

Histopathological diagnosis	Number of cases (%)
Idiopathical chronic pleuritis	33/80 (41%)
Non-small cell lung cancer	19/80 (24%)
Pleural carcinomatosis by breast cancer	11/80 (14%)
Lymphoma	4/80 (5%)
Pleural carcinomatosis by other solid malignoma	4/80 (5%)
Malignant mesothelioma	3/80 (4%)
Tuberculous pleurisy	3/80 (4%)
Small cell lung cancer	2/80 (3%)
Asbestosis	1/80 (1%)
Total	80/80 (100%)

In 79/80 cases (98.7 %, 95 %CI cannot be calculated) a diagnosis was obtained by rigid forceps biopsy. This was true in 73/80 cases (91.3 %, 95 % CI 86.0 – 96.5 %) for cryoprobe samples and in 74/80 cases (92.5 %, 95 %CI 88.6 – 97.4 %) for biopsies taken by flexible forceps. According to the assumptions made for non-inferiority, the diagnostic yield achieved with cryobiopsies was inferior to the yield rigid forceps biopsies (Difference: 12.7 %), but non-inferior to the flexible method (Difference: 6.5 %). The diagnostic yield was also different between samples harvested with rigid or flexible forceps (Difference: 10.1 %), therefore non-inferiority could neither be established for this comparison.

Analyzed per patient, in 73/80 cases (91 %) all three forceps techniques showed the concordant histopathological result. In 3/80 cases (4 %), only the samples gained by rigid forceps showed the diagnostic histology. In 1/80 case (1 %) an idiopathic pleuritis turned out to be a malignant mesothelioma after 12 month follow-up which was not detected in the thoracoscopic samples during the study.

### Complications of the procedure

No complications such as bleeding or pain occurred during the procedure in any of the biopsy techniques. Furthermore, after the procedure no complications such as empyema or prolonged fistula were noted.

## Discussion

This prospective study compared the two established biopsy techniques (rigid and flexible forceps biopsy) with the use of cryotechnique during medical thoracoscopy.

**Fig. 2:**
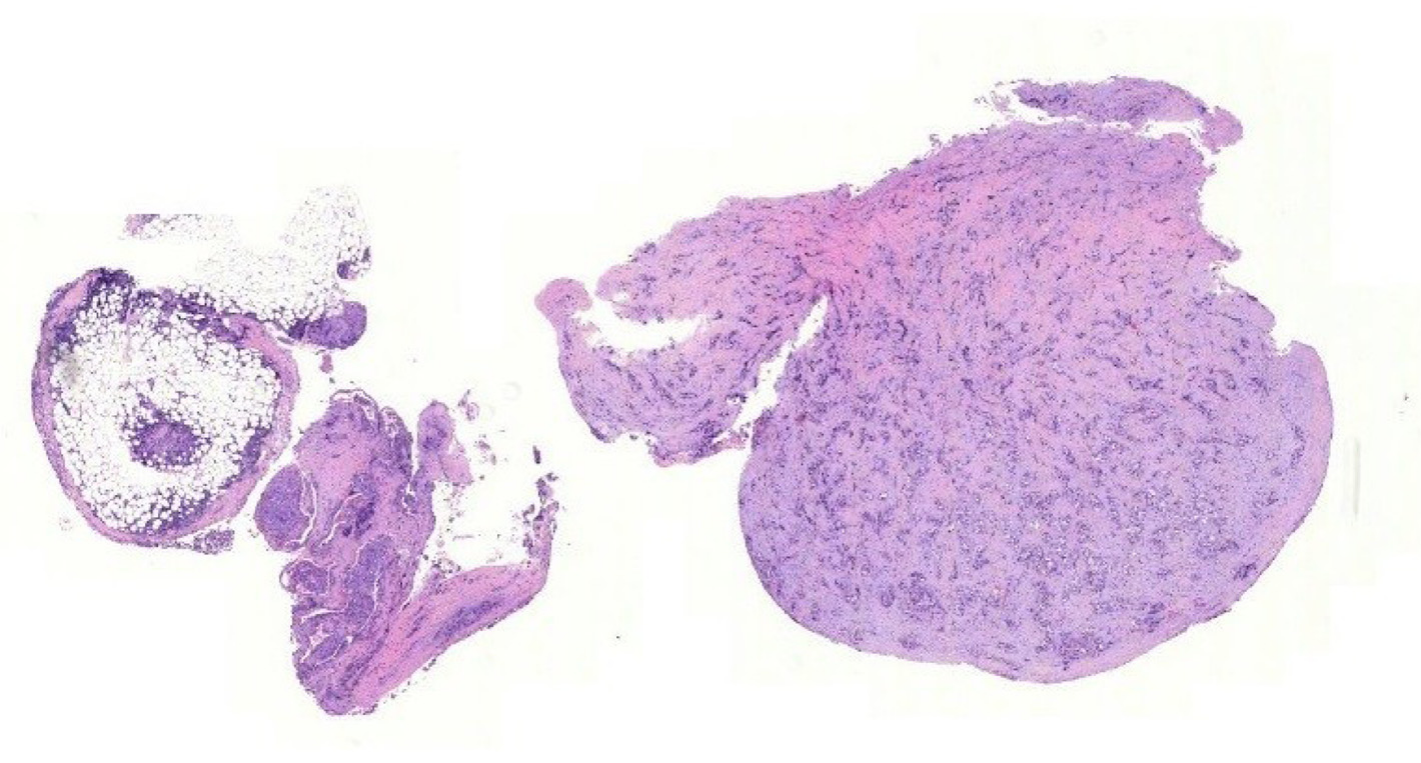
Histological comparison of biopsy techniques: diagnosis of pleural manifestation of a pulmonary, TTF1-positive adenocarcinoma

**Fig. 3:**
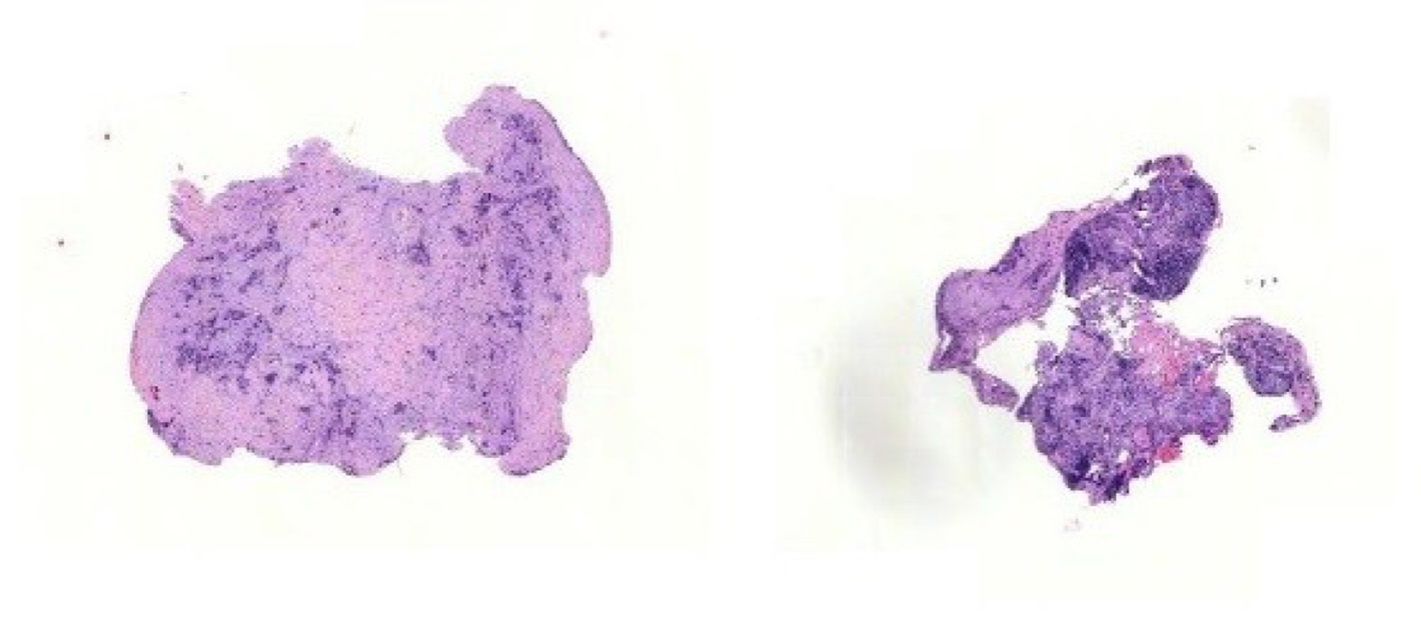
Flexible forceps biopsy of a pleural carcinomatosis of pulmonal adenocarcinoma: pleural connective tissue and desmoplastic stroma (*left side*) with infiltrates of a non-small cell carcinoma (size 3,88mm^2^)

**Fig. 4:**
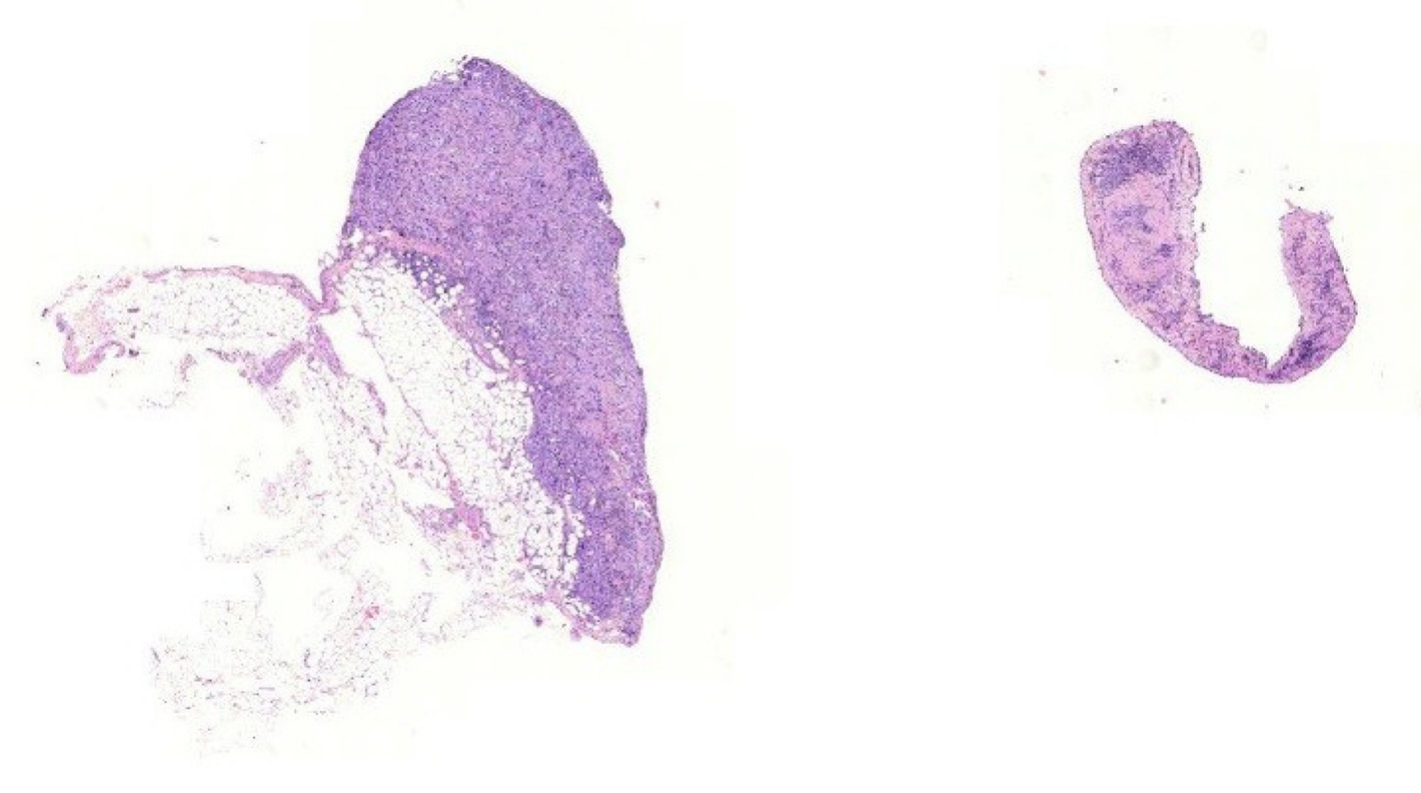
Cryobiopsy of a pleural carcinomatosis of pulmonal adenocarcinoma: pleural fat tissue (left side) and pleural connective tissue (*right side*) with infiltrates of a non-small cell carcinoma (size 11,57mm^2^)

**Fig. 5:**
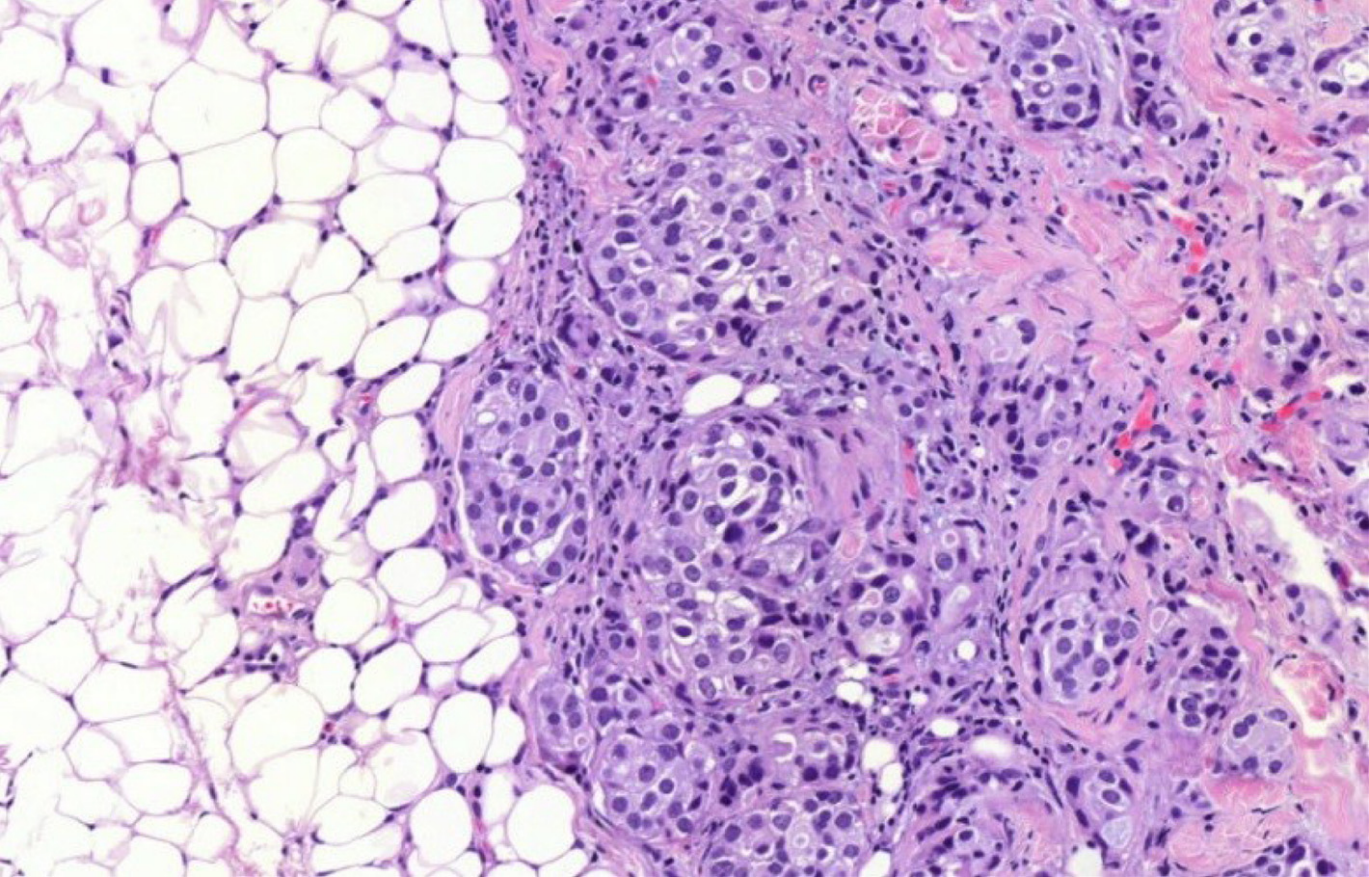
Magnification of the cryobiopsy in Fig. 3: fat tissue *(left side*), which is infiltrated by tumor nests with glandular differentiation (*right side*), a manifestation of an adenocarcinoma respect

In comparison, cryobiopsies showed a significantly larger biopsy size and depth than flexible forceps biopsies. On the other hand, rigid forceps biopsies showed, as expected, the significantly largest size and depth and the highest diagnostical yield. As cryotechnique can be used during semi-rigid thoracoscopy, and if a rigid forceps biopsy is not available, one could speculate that this inexpensive technique could overcome the problem of a too small size of flexible forceps. Furthermore, important for the pathologist was the fact that samples gained by cryotechnique showed less tissue damage in an overall very good quality (Figs 2, 3, 4 and 5) which was already described for lung biopsies [25, 26].

However, in the histopathological work-up, all three biopsy methods had a diagnostic yield of more than 90 %; in 91 % of the cases the three different techniques showed the same histologic result. In one patient, after a 12-month-follow-up that was carried out for all patients, a malignant mesothelioma was detected, that had not been found in any of the three techniques during medical thoracoscopy. This data demonstrates the non-inferiority concerning diagnostic (histological) accuracy of cryotechnique only in comparison to flexible forceps biopsy.

Furthermore, our study demonstrated the safety of the use of cryotechnique during medical thoracoscopy. No biopsy-related complications such as major bleeding or pain after tearing the probe were noted. These findings confirm the preliminary data by Rozman et al. [29], describing parietal pleural biopsies obtained by cryoprobe as safe.

In this series of 80 patients the histological diagnosis of malignant mesothelioma was only detected three times. Especially for this diagnosis the depth and quality of the biopsy is extremely important [31]. As a next step, a larger series including more patients suffering from malignant mesothelioma should be examined in a multicenter study, as this diagnosis is the most frequently missed by medical as well as surgical thoracoscopy [32]. As a hypothesis, the higher number of deep biopsies containing fatty tissue should enable to detect mesothelioma with a higher yield of cryobiopsy compared to flexible forceps biopsy [31].

## Conclusion

In summary, cryobiopsy obtained during medical thoracoscopy is a safe method with high diagnostic value, comparable to flexible forceps biopsy, but inferior to rigid forceps biopsies. Samples are smaller and less deep than rigid forceps biopsies, but significantly larger and deeper than samples gained by flexible forceps. Therefore, the use of cryobiopsy in semi-rigid thoracoscopy can not yet be generally recommended to replace rigid forceps biopsies during medical thoracoscopy.

The quantity of harvested tissue becomes increasingly important, because personalized therapy concepts especially for non-small cell lung cancer and breast cancer demand more pathologic investigations. This will favour the use of cryotechnique where rigid forceps biopsies are not available or cannot be used.

## Abbreviations

CI, confidence interval; HE, hematoxylin-eosin; mm, millimeter; SD, standard difference
